# Rheological behavior of honey adulterated with agave, maple, corn, rice and inverted sugar syrups

**DOI:** 10.1038/s41598-021-02951-3

**Published:** 2021-12-03

**Authors:** Paula Ciursa, Mircea Oroian

**Affiliations:** Faculty of Food Engineering, Stefan Cel Mare University of Suceava, Suceava, Romania

**Keywords:** Chemical engineering, Engineering

## Abstract

The aim of this study was to assess the influence of different adulteration agents (agave, maple, corn, rice and inverted sugar) on honey rheology. There was studied the influence of different percentages of adulteration agent on steady state and dynamic state rheology but also on rheology in the negative temperature domain. The authentic honey and adulterated ones behaved as a Newtonian fluid with a liquid-like behavior (Gʺ>>Gʹ). Regarding the physicochemical parameters analyzed (moisture and sugar content), significant changes depending on the adulteration agent/degree used were observed. The viscoelastical parameters (η*—complex viscosity, Gʹ —elastic modulus and Gʺ—viscous modulus) and glass transition temperature (T_g_) were predicted in function of the chemical composition (moisture content, glucose, fructose, sucrose, maltose, raffinose, trehalose, turanose, melesitose, and F/G ratio) using the PLS-R (partial least square regression). All parameters analyzed had a high regression coefficient for calibration (> 0.810) and validation (> 0.790), except for the elastic modulus.

## Introduction

Honey is a natural food product^1^ produced by bees from the nectar of flowers or honeydew^2^, being renowned for its sensorial^3^, nutritional and medicinal properties^4^. It should not contain food additives or any other exogenous substance^5^. Due to its properties, there is a high demand for honey. Thus, its adulteration with concentrated sugar syrups at a lower cost often takes place, diminishing its nutritional and medicinal benefits^6^. The most used adulteration agents are: sucrose^7^, fructose, glucose^8,9^, inverted sugar syrup^9,10^, maltose^11^, high fructose corn syrup^11,12^, beet syrup, cane syrup^13^, rice syrup^14–16^, corn syrup^10,15^, and barley syrup^15^.

Honey is a Newtonian fluid, i.e. its viscosity is not influenced by the shear rate applied^17^. The honey viscosity is influenced by the quality, processing stages of honey and temperature^18^, and the botanical origin. The botanical origin of honey influence honey viscosity due to the variation that occurs in terms of individual sugars as well as the colloidal material^19^. The fructose/glucose ratio in honey determines the crystallization rate, thus affecting both physical and rheological properties. Honey samples with a fructose/glucose ratio greater than 1.33 do not crystallize for a long time, while those with a ratio value of less than 1.11 crystallize very easily. There are also other factors that act as seed crystals such as pollen, other carbohydrates, air bubbles, moisture content^20^. Glucose can crystallize as α-d-glucose monohydrate at temperatures below 50 °C. The other two forms, anhydrous α-d-glucose and anhydrous β-glucose, are stable at temperatures between 50 and 80 °C. Natural honey has a Newtonian behavior, and its rheological properties are influenced by temperature, while crystallized honey has a non-Newtonian behavior^21^. The presence of compounds with high molecular weight such as proteins or polysaccharides (dextran) in honey composition can cause non-Newtonian behavior^22^. Polymers with a concentration of 2% melesitose show a non-Newtonian behavior, and in some honey it was reported in a percentage of 11%^23^.

The addition of molasses in honey led to the change of the Newtonian behavior present in pure honey to a non-Newtonian pseudoplastic behavior present in adulterated honey in different concentrations^24^. Yilmaz et al. reported that the shear stress of honey samples adulterated with saccharose and fructose syrup decreased depending on the degree of adulteration. Also, the addition of syrup led to a decrease in viscosity, viscous and elastic moduli values^21^.

Nguyen et al. studied the viscoelastic properties at temperatures below zero degrees for pure Tulsi honey. There was an increase in parameters Gʹ (storage modulus) and Gʺ (loss modulus) known as the glass transition, with the viscous component dominating the elastic component. Around the temperature of − 42 °C, the two moduli intersected with the production of a dominant elastic response, thus taking place in the glassy state, a state in which the physicochemical reactions are limited^25^. Honey is considered a high-solid material and presents a glass transition temperature which is influenced by the chemical composition and it is used as a parameter for predicting the quality and stability; above this temperature the product has a rubbery and/or a melt state which may lead to a structural change of the material which includes collapse, stickiness, caking and fusion^26^.

Starting from this aspect, in this study are presented the rheological properties of authentic and adulterated Romanian acacia honey with different types of syrups (agave, maple, corn, rice and inverted sugar). For this purpose, steady and dynamic state, as well as rheology in the negative temperature domain were analyzed. To our knowledge, there is no study that reported on the use of rheology at temperatures below zero degrees as a tool for the detection of adulterated honey.

## Materials and methods

### Materials

The authentic acacia Romanian honey was purchased from a local beekeeper (Suceava County). The syrups used in honey adulteration were purchased from the commercial market: agave (Clarks, Mexico), maple (BioLogistic & Distribution Partener, Canada), rice (Panaisia de Hadels GMBH Importing company, Korea) and corn (Daesang Europe B.V. Importing company, Korea). Inverted sugar syrup was obtained by hydrolysis of sucrose with the addition of citric acid. Honey adulteration was done by adding syrup in percentages of 5%, 10%, 20% and 50% into the authentic honeys.

### Methods

#### Chemical composition of authentic and adulterated honeys

##### Moisture content

The moisture content is the value determined from the refractive index of honey by conversion using a standard table (Chataway table). The samples were previously liquefied at 50 °C for 24 h. A drop of honey sample was placed on an Abbé refractometer (Leica Mark II Plus, Germany), which was previously calibrated with distilled water. The measurement was performed at 20 °C, and the final result obtained was expressed as a percentage (%).

##### Sugar content

Sugar content determination was based on the method published by Bogdanov and Baumann (1988) using a high performance liquid chromatograph (Shimadzu, Japan) with a refractive index detector (RID-10A)^27^. The separation was performed on a Phenomenex Luna® Omega 3 µm SUGAR 100 Å LC (150 × 4.6 mm) column. The standard substances used were: glucose, fructose, sucrose, maltose, trehalose, melesitose, and raffinose.

Sample preparation was carried out as follows: 5 g of honey were dissolved in 40 mL of distilled water and transfer into a 100 ml volumetric flask, it was added 25 mL of methanol. The mixture was mixt and the flask was filled up to mark with distilled water. The final solution was filtered into vials through 0.45 μm membrane filters. Samples were injected in a volume of 10 μl. The samples were analyzed in duplicate.

The mobile phase was a mixture of acetonitrile and water in a proportion of 80:20 and the flow rate was 1.3 ml/min. The detector and column temperature were set at 30 °C. The concentration of the sugars in honeys was determined using the calibration curve for each sugar. The samples were analyzed in duplicate.

#### Rheological properties

The dynamic rheological properties and rheology in negative temperature region were performed using a Mars 40 rheometer (Thermo Haake, Germany). A parallel plate system of 40 mm diameter was used at a gap of 1 mm. All measurements were done in triplicate.

##### Dynamic state

The rheological measurements were performed at temperatures of 5 °C, 10 °C, 20 °C, 30 °C and 40 °C. The measurements were performed in triplicate under the same conditions for each sample. After the sample was loaded, it took a waiting period (5 min) for the sample to reach the set temperature. To determine the linear viscoelastic region, the frequency measurements were made at 1 Hz. Then, the frequency range was between 0.1 and 10 Hz at a tension of 1 Pa (which was in the linear viscoelastic region for all the samples). Rheowin Job software (v.4.86, Haake) was used to obtain the experimental data but also to calculate the rheological parameters (complex viscosity—η*, storage or elastic mode— Gʹ and loss or viscous modulus— Gʺ).

The viscoelastical parameters (elastic and loss modulus) were submitted to non-linear regression using the power law function^28^

1 $$G^{\prime}={K}^{\prime}{w}^{n{{\prime}}}$$

2 $$G^{\prime\prime}=K^{\prime\prime}w^{n\prime\prime}$$

where Gʹ—elastic modulus, Gʺ—loss modulus, Kʹ—elastic intercept, Kʺ—loss intercept, nʹ—elastic slope, nʺ—loss slope.

##### Rheology in negative temperature region

The analyzed sample was allowed to rest for 5 min to reach a temperature of 20 °C, then the temperature was lowered to − 15 °C. The measurements were performed from – 15 to − 40 °C, following the intersection of the two moduli (storage and loss). The temperature where the two moduli were crossing is named glass transition temperature. The rheometer was connected to a cooling bath thermostat (Huber—Pilot ONE, Germany) to achieve rapid cooling. The recirculation solution used was ethanol (96%). The measurements were performed in triplicate under the same conditions for each sample.

### Statistical analysis

The results were submitted to analysis of variance (ANOVA) using XLSTAT trial version (Microsoft, Charlotte, NC, USA). Fisher’s least significant difference (LSD) procedure was used at the 95% confidence level. The partial least square regression (PLS-R) was made using Unscrambler X 10.1 (Camo, Norway).

## Results and discussion

### Chemical composition

Table 1 presents the values obtained for the moisture and sugar content of the authentic honey sample, adulteration agents and adulterated samples. Authentic honey had a moisture content of 15.96%. Fructose was the major chemical compound found in the acacia honey, followed by glucose; the sucrose content was lower than 2% which means that the sample was a mature one and neither an adulterated one^29^. The ratio F/G was higher than 1 which confirms the liquid state of honey. Another substance which is considered to contribute to the liquid state was trehalose that was reported in a concentration higher than 1%^30^. The results of the chemical composition of acacia honey were similar to those reported in other studies^29,31^.

**Table 1:** Chemical composition of acacia honey and honey adulterated with corn syrup (C), rice syrup (R), inverted sugar syrup (IS), agave syrup (A) and maple syrup (M).

Sample		Moisture content (%)	Fructose (%)	Glucose (%)	Sucrose (%)	Turanose (%)	Maltose (%)	Trehalose (%)	Melesitose (%)	Raffinose (%)	F/G ratio (%)
Acacia honey		15.96	37.19	25.93	0.45	0.17	2.19	1.15	1.32	0.51	1.42
Corn syrup (C)		22.02	0.05	1.55	0	0	0	0	35.03	0.11	0.03
Rice syrup (R)		17.31	0.26	14.54	0.14	0	0	0	36.19	0	0.02
Inverted sugar syrup (IS)		17.74	34.62	30.93	0.23	1.24	0	0	0.04	0.06	1.11
Agave syrup (A)		22.77	47.22	16.40	0.50	0.52	0	0.10	0	0	2.85
Maple syrup (M)		32.19	2.11	2.96	56.20	0	0	0	0.03	0	0.70
Honey adulterated with C	5%	16.26	35.33	24.71	0.42	0.16	2.08	1.09	3.01	0.49	1.42
	10%	16.56	33.47	23.49	0.40	0.15	1.97	1.04	4.69	0.47	1.41
	20%	17.17	29.76	21.05	0.36	0.13	1.75	0.92	8.06	0.43	1.40
	50%	18.99	18.62	13.74	0.22	0.08	1.09	0.58	18.18	0.31	1.34
Honey adulterated with R	5%	16.03	35.34	25.36	0.43	0.16	2.08	1.09	3.06	0.49	1.38
	10%	16.09	35.50	24.79	0.42	0.15	1.97	1.04	4.81	0.46	1.34
	20%	16.23	29.80	23.65	0.38	0.13	1.75	0.92	8.29	0.41	1.25
	50%	16.63	18.73	20.24	0.29	0.08	1.09	0.58	18.76	0.26	0.92
Honey adulterated with IS	5%	16.05	37.06	26.18	0.44	0.22	2.08	1.09	1.26	0.49	1.40
	10%	16.14	36.93	26.43	0.42	0.28	1.97	1.04	1.19	0.47	1.38
	20%	16.32	36.67	26.93	0.40	0.38	1.75	0.92	1.06	0.42	1.35
	50%	16.85	35.90	28.43	0.34	0.70	1.09	0.58	0.68	0.28	1.25
Honey adulterated with A	5%	16.30	37.69	25.45	0.45	0.19	2.08	1.10	1.25	0.49	1.47
	10%	16.64	38.19	24.98	0.45	0.20	1.97	1.05	1.19	0.46	1.51
	20%	17.32	39.19	24.02	0.46	0.24	1.75	0.94	1.06	0.41	1.62
	50%	19.36	42.21	21.17	0.47	0.34	1.09	0.63	0.66	0.26	1.97
Honey adulterated with M	5%	16.77	35.43	24.78	3.23	0.16	2.08	1.09	1.26	0.49	1.42
	10%	17.58	33.68	23.63	6.02	0.15	1.97	1.04	1.19	0.46	1.41
	20%	19.21	30.17	21.33	11.60	0.13	1.75	0.92	1.06	0.41	1.40
	50%	24.08	19.65	14.44	28.32	0.08	1.09	0.58	0.68	0.26	1.35

The adulteration agent compositions were very different from the honey with the exception of inverted sugar; corn syrup had a high concentration of melesitose, rice syrup has a high concentration of melesitose and glucose, agave syrup had a high concentration of fructose and glucose, while agave syrup has a high concentration of sucrose. The inverted sugar composition was similar to that of honey, with a fructose concentration higher than the glucose concentration. The results were in agreement with the literature^31^.

All adulteration agents produced an increase in moisture content, and the maximum allowed limit of 20% was exceeded only in the case of honey adulterated with 50% maple syrup (24.08%). A similar high moisture content (24.01%) was also associated with adulterated honey^32^. El-Bialee and Sorour showed that a moisture content between 20.7 and 39.6% can signal an adulteration with starch, glucose or water^24^. Honey adulterated with a saturated sugar solution also showed high moisture values (19.30%) compared to authentic honey (16.70%) from Pakistan^33^. In general, the average glucose content is 26.3%^34^, which is a value very close to that obtained for the sample used in this study. Regarding the fructose content, other researchers reported a higher content^34–36^. The addition of maple syrup, corn and rice to authentic honey produced a significant decrease in fructose and glucose content. A decrease in the concentration of fructose and glucose was also observed in honey adulterated with sucrose syrup^37^. Honey adulterated by adding inverted sugar syrup had a fructose and glucose content close to that present in authentic honey. The sucrose content was low in both authentic honey and honey adulterated samples with corn, inverted sugar, rice and agave syrups. Only the maple syrup produced an increase from 0.45% (authentic honey) to 28.32% in honey adulteration with 50% syrup, the maximum allowed value of 5% being exceeded 5.6 times. A decrease in maltose content occurred in all adulterated samples, which was not detected in any adulteration agent. In addition, all adulteration agents also decreased the content of turanose (was present only in agave syrup) and raffinose. As for the melesitose content, it increased approximately 14 times in honey adulterated with 50% corn syrup (18.18%) and rice syrup (18.76%) compared to authentic honey (1.32%).

### Honey rheology

**Figure 1:**
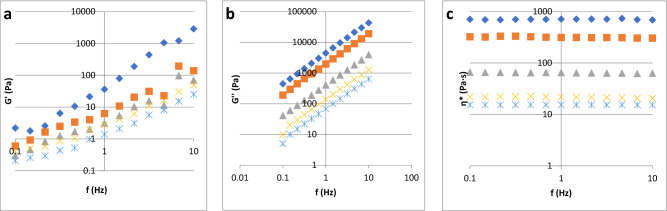
Acacia honey viscoelastical parameter evolution in function of temperature: (**a**) elastic modulus, (**b**) loss modulus, (**c**) complex viscosity: rhombus—5 °C, square—10 °C, triangle—20 °C, cross—30 °C, sign of multiplication—40 °C.

**Figure 2:**
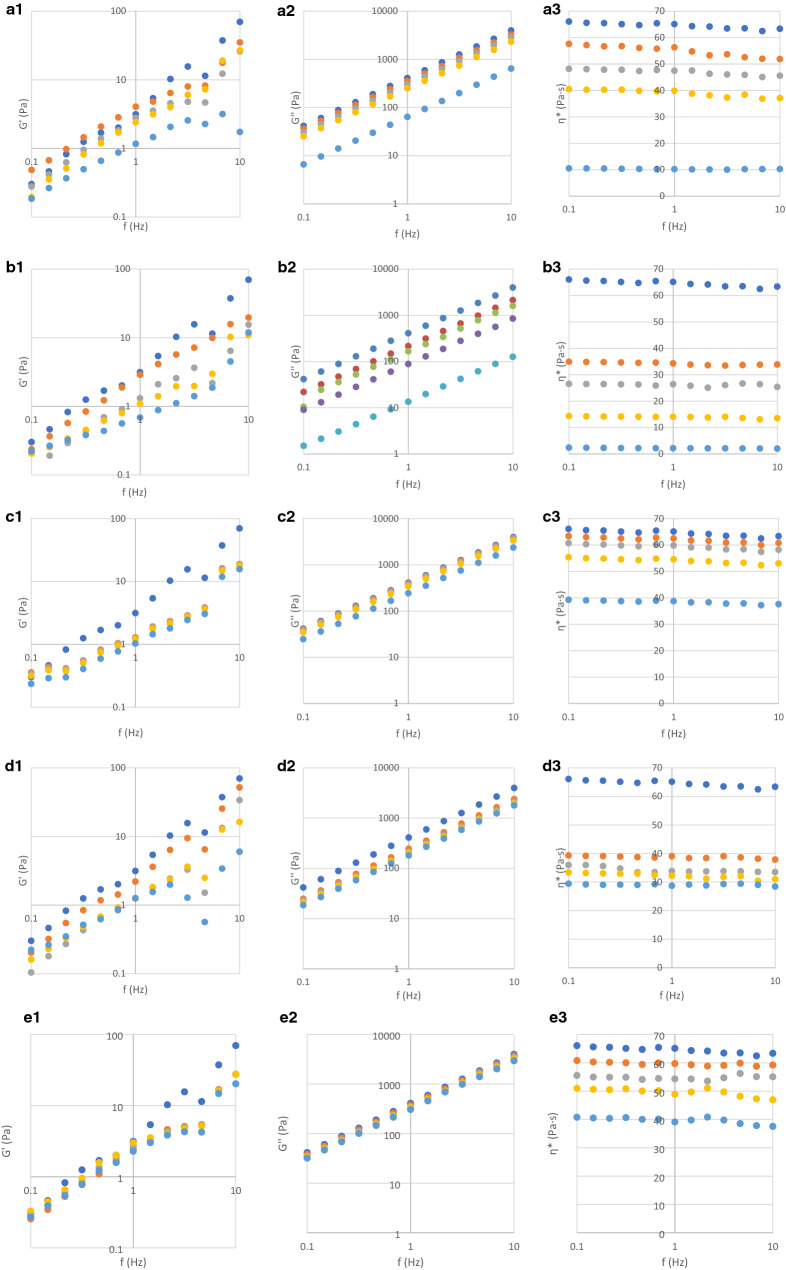
Dynamic rheological behaviour of honey (1—elastic modulus, 2—loss modulus, 3—complex viscosity) adulterated with: (**a**) agave syrup, (**b**) maple syrup, (**c**) inverted sugar, (**d**) corn syrup, (**e**) rice syrup. Dark blue dot: authentic; orange dot: 5% adulteration; grey dot: 10% adulteration; yellow dot: 20% adulteration; light blue dot: 50% adulteration.

In Fig. 1 is presented a typical dynamical rheogram for honey (elastic modulus, viscous modulus and complex viscosity), while in the Table 2 is presented the dynamic viscosity and curve fitting of elastic modulus and loss modulus of acacia honey and adulterated samples with different syrups. As it can be observed in the Fig. 1, the acacia honey behaved as a Newtonian liquid irrespective of the temperature and frequency applied. In Fig. 2 are presented the evolution of the viscoelastical parameters with adulteration, and, as it can be observed, the Newtonian trend is observed in all the samples. The Newtonian behavior is presented as a viscosity plateau irrespective of the frequency applied to the testing material; in the scientific literature a similar behavior was reported for honey^17,38–42^. The dynamic viscosity (Table 2) of the authentic honey was 65.12 Pa s, and it can be observed that the addition of the adulteration agents into authentic honey led to a decrease of the parameter value (from 65.12 Pa s for the authentic honey to 2.15 Pa s in the case of honey adulterated with 50% of maple syrup);this reduction can be attributed to the modification of the chemical composition of the samples. The addition of agave and maple syrups decreased the most the dynamic viscosity, followed by corn syrup. In the case of inverted sugar and rice syrup, the addition of 5%, 10% and 20% lead to a slow decreasing of the viscosity up to 23% (honey adulterated with 20% rice syrup), while the addition of 50% syrup led to a decreasing of the viscosity up to 59%. Yilmaz et al. (2014) observed a decrease in the value of dynamic viscosity in honey samples adulterated with fructose and sucrose^21^. The addition of agave, maple, inverted sugar, corn and rice syrups decreased significantly the dynamic viscosity of honey (P < 0.05).

**Table 2:** Dynamic viscosity and parameters of curve fitting of elastic modulus and loss modulus of acacia honey and adulterated samples with different syrups.

Sample	Dynamic viscosity (Pa s)	*G*ʹ			*G*ʺ			*T*_*g*_ (°C)
		*K*ʹ	*n*ʹ	*R*^2^	*K*″	*n*″	*R*^2^	
Acacia	65.12	1.68	0.83	0.92	405.35	0.99	1	− 17.12
A_agave 5%_	56.32	3.54	0.83	0.97	345.21	0.97	1	− 19.08
A_agave 10%_	47.47	2.31	0.85	0.96	295.19	0.98	1	− 20.18
A_agave 20%_	39.87	2.25	0.98	0.98	245.15	0.97	0.99	− 22.76
A_agave 50%_	10.20	0.95	0.58	0.9	64.43	0.99	1	− 29.05
A_maple5%_	34.30	2.48	0.95	0.99	214.89	0.99	1	− 20.72
A_maple 10%_	26.38	3.99	0.42	0.02	184.07	0.94	0.96	− 23.39
A_maple 20%_	14.04	1.16	0.83	0.95	87.57	0.98	0.99	− 28.96
A_maple 50%_	2.15	0.87	0.73	0.89	13.63	0.97	0.99	− 36.14
A_inverted sugar 5%_	62.48	1.63	0.83	0.92	388.94	0.98	1	− 17.60
A_inverted sugar 10%_	59.84	1.58	1.17	0.46	372.54	0.98	1	− 18.34
A_inverted sugar 20%_	54.57	1.49	0.84	0.92	339.73	0.98	1	− 19.70
A_inverted sugar 50%_	38.74	1.22	0.87	0.94	241.3	0.98	1	− 22.91
A_corn 5%_	39.01	1.41	0.79	0.94	243.17	0.99	1	− 18.33
A_corn 10%_	33.85	1.23	1.03	0.9	214.98	0.98	0.99	− 19.74
A_corn 20%_	32.19	1.31	0.93	0.95	202.14	0.98	0.99	− 21.83
A_corn 50%_	28.68	0.91	0.57	0.76	182.48	0.99	1	− 26.76
A_rice 5%_	58.72	2.20	0.94	0.97	392.21	0.98	0.99	− 17.24
A_rice 10%_	54.34	1.86	0.75	0.93	344.23	1	0.99	− 17.91
A_rice 20%_	48.89	2.06	0.86	0.96	331.64	0.98	0.99	− 18.79
A_rice 50%_	39.91	2.47	0.85	0.95	324.11	0.99	1	− 20.55

The viscoelastical parameters of honey and adulterated samples were influenced strongly by the frequency (Figs. 1, 2); it can be observed a positive correlation between the frequency applied and the moduli magnitude. In Figs. 1 and 2 it can be observed that the loss modulus is much higher than the elastic modulus (Gʺ >> Gʹ), behavior that is typical to the materials which are similar to a liquid-like macromolecular ones^17,43^. In Table 2 are presented the correlation coefficients for curve fitting of elastic modulus and loss modulus of acacia honey and adulterated samples with different syrups; all samples behaved as liquid-like materials because Kʺ (13.63–405.35)>> Kʹ (0.87–3.54). The values of Gʹ and Gʺ for all the analyzed samples had a high dependence to the frequency applied (nʹ = 0.42–1.17, nʺ = 0.94–1.00). The Kʹ, Kʺ, nʹ and nʺ are in the same range with those reported in the literature^43,44^. The magnitude of the two intercepts increased with the increasing water content and decreased with the increasing temperature.

#### Honey rheology in negative temperatures

**Figure 3:**
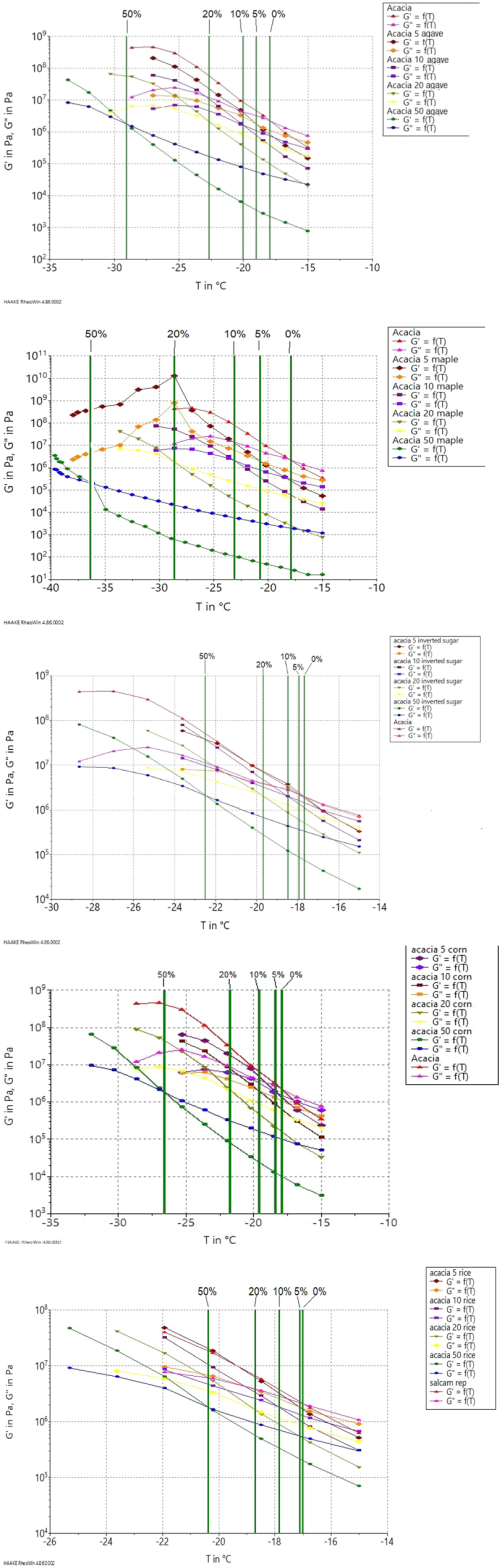
Rheological behaviour of honey adulterated with syrups in the negative temperatures. Green line: crossover of loss modulus with elastics modulus.

As it was observed in the Figs. 1 and 2, the rheological behavior of honey at positive temperatures was liquid-like behavior, where the loss modulus Gʺ was much higher than the elastic modulus Gʹ^45^. In order to evaluate the rheological behavior of the samples in the negative temperatures, the samples were measured at temperatures from – 15 to − 40 °C (Fig. 3). It can be observed a very sharp increase of the moduli from 10^3^ up to 10^9^ Pa with the decrease of the temperature applied.

This negative temperature applied to the rheological measurements is known as the glass transition region where the viscous components are becoming less important and the elastic components increase more than the first one (Gʹ  > Gʺ); this is also called glassy state, a state in which physicochemical reactions are limited due to long-range molecular movements^25^. The crossing point where the two components have the same magnitude is known as glass transition point which correspond to a glass transition temperature. As it can be observed in Fig. 3, the acacia honey glass transition temperature was − 17.12 °C, and decreased with the increasing of the adulteration percentage until − 36.14 °C (honey adulterated 50% with maple syrup) (Table 2 and Fig. 3). In Fig. 3 it can be observed the evolution of the T_g_ for each type of adulteration agent. In the case of inverted sugar and rice addition to the authentic honey there was not observed a high decrease of the glass transition temperature, while in the case of honey adulterated with high concentration of maple, corn or agave syrups the difference was notable. According to the Pearson correlation there was observed a strongly negative correlation between T_g_ and moisture content (r = − 0.961*) and sucrose (r = − 0.782*), and a strong positive correlation between T_g_ and fructose (r = 0.436*), glucose (r = 0.743*), maltose (r = 0.694*), trehalose (r = 0.687*), and raffinose (r = 0.711*).

#### Rheological parameters prediction using PLS-R

**Figure 4:**
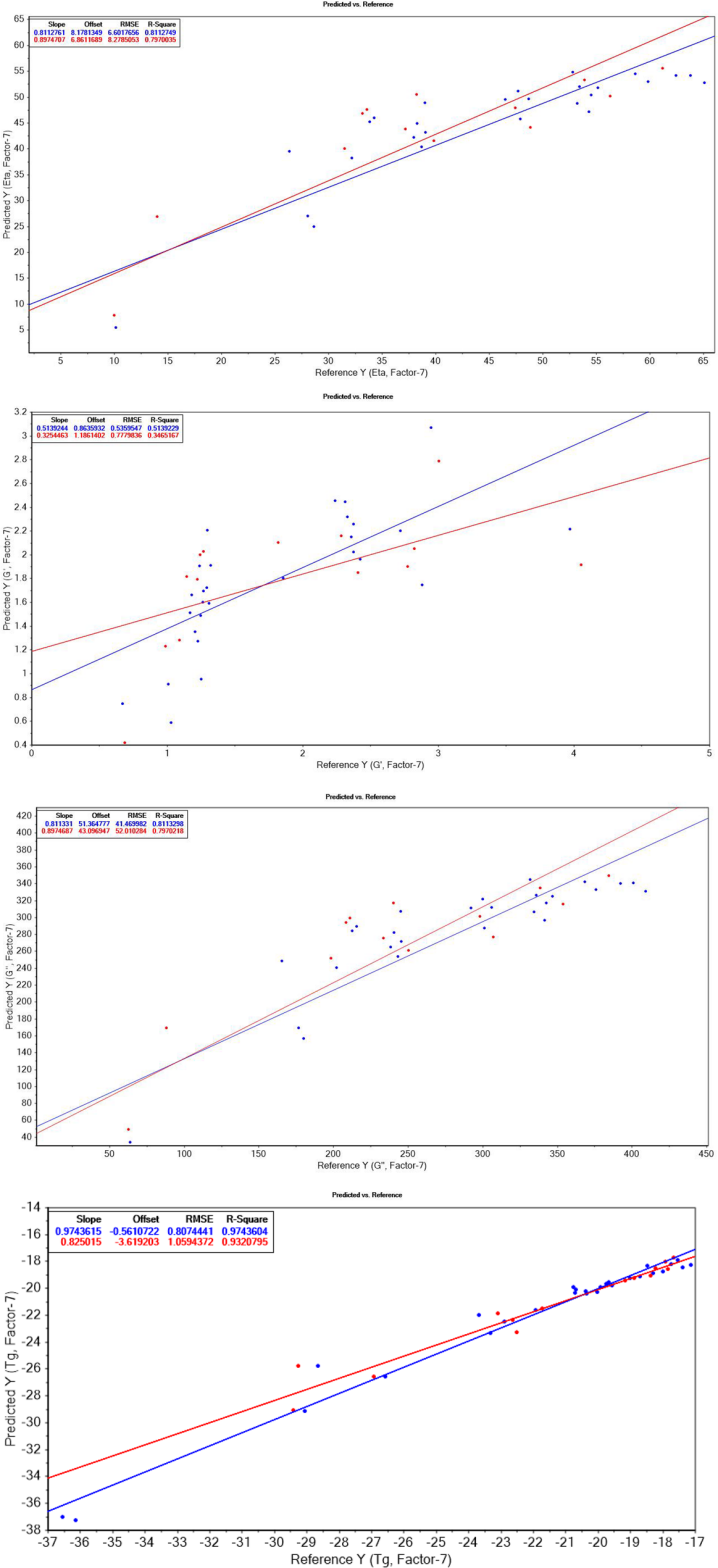
PLS-R model for the prediction of rheological parameters (dynamic viscosity (eta), Gʹ and Gʺ) and T_g_: experimental vs predicted values.

The PLS-R model was used for the prediction of rheological parameters (η, Gʹ and Gʺ) and glass transition temperature (T_g_) in function of chemical composition (moisture content, fructose, glucose, sucrose, turanose, maltose, trehalose, melesitose, raffinose and F/G). The data were split into two categories: calibration (66.6% of the samples) and validation (33.3% of the samples) in order to achieve the desired model. The number of maximum factors for the prediction of the parameters was set at 7. Different statistical parameters were used to verify the predicted model: slope, offset, RMSE, and regression coefficient. For this purpose, the data was divided, as follows: 66% for the calibration stage and 33% for the validation stage. The results of the calculations performed using the data obtained for the calibration and validation stages for the parameters model (slope, offset, RMSE, R2), as well as the model parameters that reached the best statistical results are presented in Table 3. With the exception of elastic modulus, all the other parameters analyzed had high regression coefficient for calibration (> 0.810) and validation (> 0.790). The loss modulus and dynamic viscosity had the same regression coefficient, and this can be attributed to the liquid-like state of honey where the viscous behavior is much bigger than the elastic behavior, honey being a Newtonian liquid^43^. The experimental vs predicted values of the parameters analyzed are presented in Fig. 4.

**Table 3:** Statistical results of the rheological parameters and T_g_ prediction using the partial least square regression based on the chemical composition.

Parameter	No. factors	Calibration				Validation			
		Slope	Offset	RMSE	R^2^	Slope	Offset	RMSE	R^2^
Dynamic viscosity (Pa s)	7	0.811	8.178	6.601	0.811	0.897	6.861	8.278	0.797
Gʹ (Pa)	7	0.513	0.863	0.535	0.513	0.325	1.186	0.777	0.346
Gʺ (Pa)	7	0.811	51.364	41.469	0.811	0.897	43.096	52.010	0.797
T_g_ (°C)	7	0.974	− 0.561	0.807	0.974	0.825	− 3.619	1.059	0.932

### Principal component analysis

**Figure 5:**
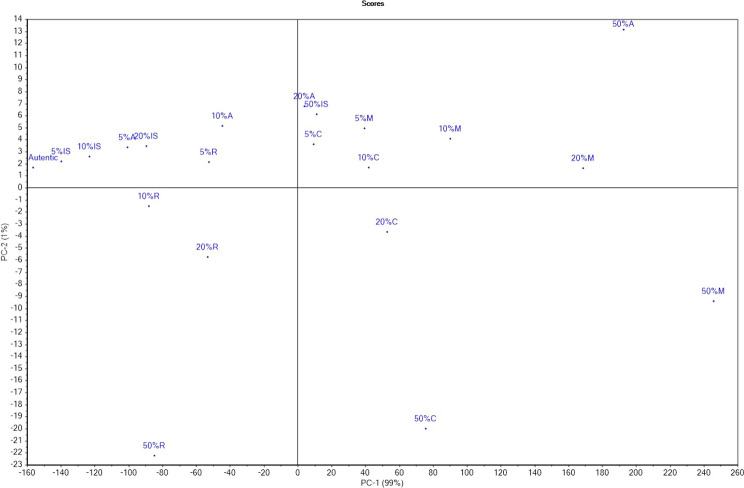
Principal component scores of honey and honey adulterated: *IS* inverted syrup, *C* corn syrup, *M* maple syrup, *A* agave syrup, *R* rice syrup.

**Figure 6:**
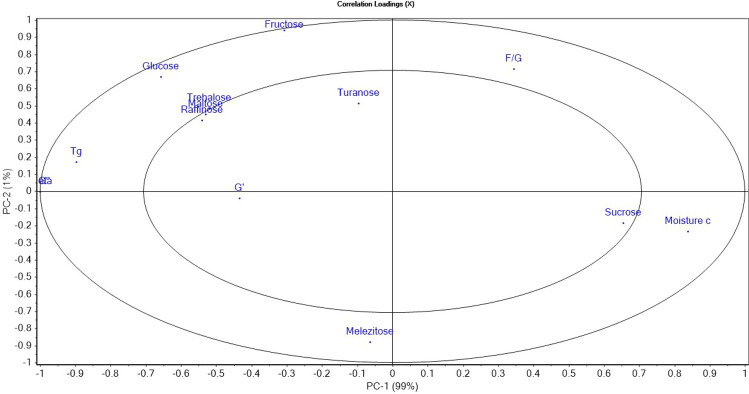
Principal component analysis loadings of the parameters studied.

Figures 5 and 6 show PCA scores and loadings. The first component (PC-1) represented 99% of the variance and the second component (PC-2) 1%, totaling 100% of the initial variability. As can be seen, the authentic sample as well as the adulterated ones with 5%, 10% and 20% inverted sugar syrup, 5% and 10% agave syrup and 5% rice syrup are placed in the same quadrant, which means that adulteration agents mimic the properties of honey very well; the fact that the PC1 represents 99% of the total variance it is possible that samples placed in the same quadrant to be different. The samples adulterated with corn syrup and maple syrup are in different quadrants due to their composition. Moreover, there was a similarity between the samples adulterated with 50% inverted sugar syrup, 20% and 50% agave syrup, 5% and 10% corn syrup and 5%, 10% and 20% maple syrup. According to PCA-loadings, the honey samples were mostly influenced by the following parameters: content of fructose, glucose, melesitose, F/G ratio, moisture content, but also viscosity (η), loss modulus (Gʺ) and glass transition temperature (T_g_). In addition, the overlap of the rheological parameters viscosity-loss modulus indicated a strong correlation.

## Conclusions

Rheological parameters were successfully used in the detection of honey adulterated with agave, maple, corn, rice, and inverted sugar syrups in different percentages. The prediction of the rheological parameters was achieved using the PLS-R model depending on the chemical composition of the samples. Regarding the physicochemical parameters analyzed (moisture and sugar content), they showed significant changes depending on the adulteration agent/degree used. The addition of the adulteration agents into the authentic honey led to a decrease of dynamic viscosity from 65.12 Pa s for the authentic honey to 2.15 Pa s in the case of honey adulterated with 50% of maple syrup. The rheological behavior of honey at positive temperatures indicates much higher values of the viscous modulus compared to the elastic one. At negative temperatures, below − 15 °C, there was an increase in the values of the elastic modulus with a domination of the viscous modulus, taking place a crossover of the two moduli, known as the glass transition point. Thus, acacia honey glass transition temperature was − 17.12 °C, and decreased with the increase of the adulteration percentage until − 36.14 °C in honey adulterated 50% with maple syrup. The most notable differences were in the case of honey samples adulterated with high concentrations of maple, corn and agave syrups.

## Data Availability

The data used to support the findings of this study are available from the corresponding author upon request.
